# The methods and advances of adaptive immune receptors repertoire sequencing

**DOI:** 10.7150/thno.61390

**Published:** 2021-08-19

**Authors:** Hongmei Liu, Wenjing Pan, Congli Tang, Yujie Tang, Haijing Wu, Akihiko Yoshimura, Yan Deng, Nongyue He, Song Li

**Affiliations:** 1 Hunan Key Laboratory of Biomedical Nanomaterials and Devices, Hunan University of Technology, Zhuzhou 412007, China.; 2 State Key Laboratory of Bioelectronics, Southeast University, Nanjing 210096, China.; 3 Department of Dermatology, Second Xiangya Hospital, Central South University, Hu-nan Key Laboratory of Medical Epigenomics, Changsha, Hunan, China.; 4 Department of Microbiology and Immunology, Keio University School of Medicine, Tokyo, Japan.

**Keywords:** adaptive immune repertoire sequencing, T or B cell receptors, high-throughput sequencing, bioinformatics

## Abstract

The adaptive immune response is a powerful tool, capable of recognizing, binding to, and neutralizing a vast number of internal and external threats via T or B lymphatic receptors with widespread sets of antigen specificities. The emergence of high-throughput sequencing technology and bioinformatics provides opportunities for research in the fields of life sciences and medicine. The analysis and annotation for immune repertoire data can reveal biologically meaningful information, including immune prediction, target antigens, and effective evaluation. Continuous improvements of the immunological repertoire sequencing methods and analysis tools will help to minimize the experimental and calculation errors and realize the immunological information to meet the clinical requirements. That said, the clinical application of adaptive immune repertoire sequencing requires appropriate experimental methods and standard analytical tools. At the population cell level, we can acquire the overview of cell groups, but the information about a single cell is not obtained accurately. The information that is ignored may be crucial for understanding the heterogeneity of each cell, gene expression and drug response. The combination of high-throughput sequencing and single-cell technology allows us to obtain single-cell information with low-cost and high-throughput. In this review, we summarized the current methods and progress in this area.

## Introduction

**Figure 1:**
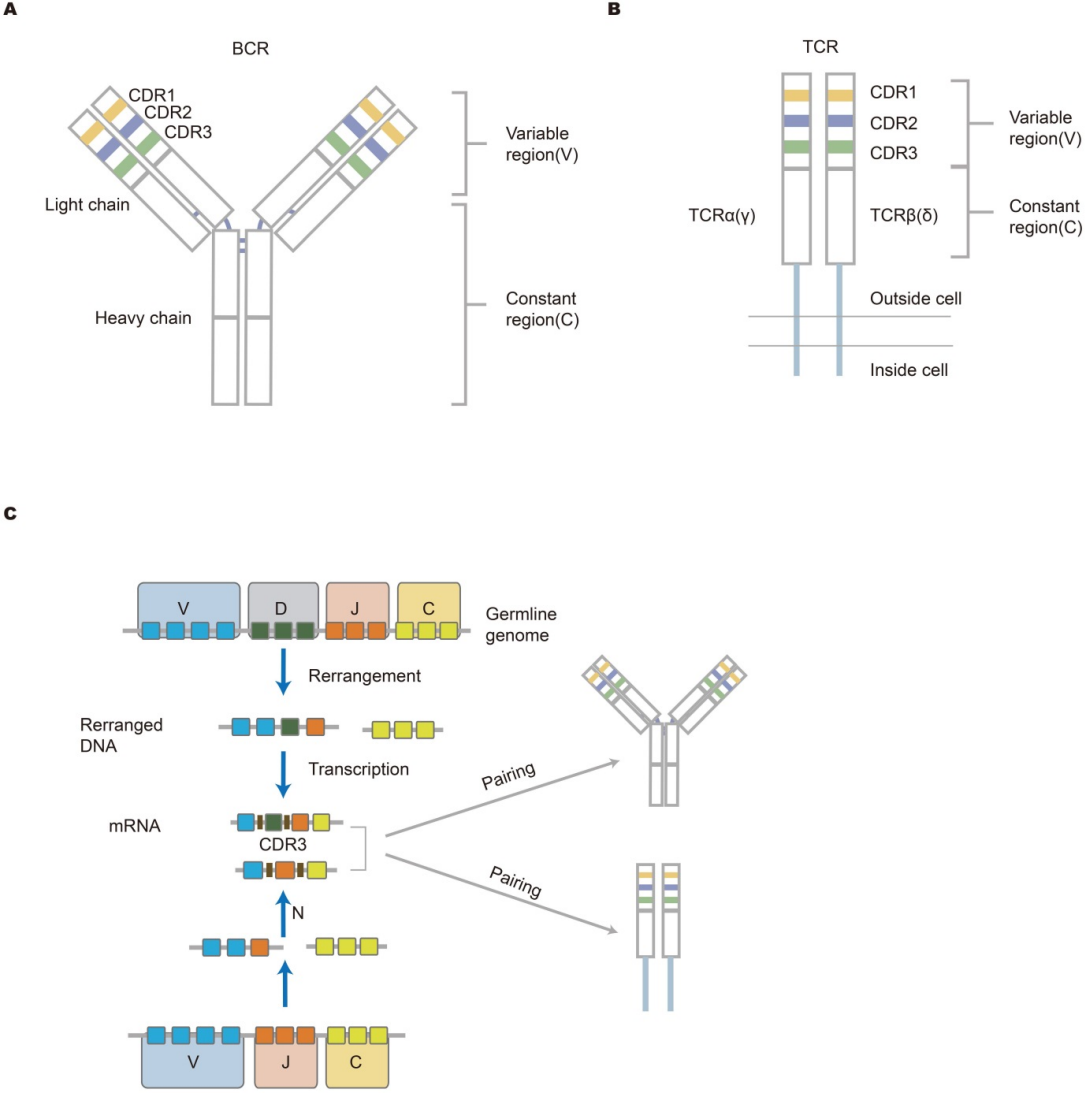
** The structure of antigen-specific lymphocyte receptors and the generation of diversity. (A, B)** The structure of BCR and TCR. The heavy and light chains of antibodies are shown, and they are connected by disulfide bonds (bold blue line); TCR that is across the cell membrane is a heterodimer comprised of αβ chains or γδ chains. The upper part is the variable (V) region composed of V(D)J gene in the figure. The V region is composed of CDR regions and FR domains. CDR1, CDR2, and CDR3 are shown in different colors (yellow, blue, and green, respectively). The lower part (white area) is a constant region that is conservative. The structure of lymphocyte receptors shown here contributes to explaining how immune response occurs. **(C) The mechanism of lymphocyte receptors repertoire diversity.** During the development of lymphocytes, BCR heavy chains or TCR β(δ) chains suffer from the rearrangement of VDJ genes, while the IgL chain or α(γ) chain lack D gene in the rearrangement. Afterward, the rearranged V-DJ or V-J sequences are linked to the C gene fragments. Finally, two independent chains are assembled into unique receptor proteins. Germline gene V(D)JC undergoes rearrangement and insertion and deletion of nucleotides, resulting in the diversity of the receptor library 16.

The immune system protects the human body from the harm of pathogens. This protection depends mainly on an immune response which primarily is induced by a vast number of lymphocyte cell receptors, T-cell receptors (TCRs) and B-cell receptors (BCRs). Immune receptors are an important structural domain where antigens bind. There are a total of seven chains forming receptors. T cells include αβ T cells that are coded by alpha and beta chains or γδ T cells that are coded by gamma and delta chains. B cell receptors are coded by heavy and kappa chains or heavy and lambda chains. There are also dual alpha 1, 2 and dual gamma T cells 3, 4. These receptors contribute to determining antigen specificity 5, 6. The total of unique TCRs and BCRs make up the immune repertoire in one individual at any given moment, which reflects the ability of the immune system to respond to toxins or foreign substances. Each unique antigen-specific receptor is comprised of variable (V) gene, diversity (D) gene, joining (J) gene, and constant (C) gene. The combination of these gene segments determines the specificity and diversity of lymphocytes. TCRs and BCRs have three complementarity determining regions, including CDR1, CDR2 and CDR3. The CDR3 region is the most variable portion of the antigen-binding site 7. In addition to recombination diversity, the addition and deletion of nucleotides that occur at the junction of gene fragments contribute to junctional diversity 8. B cell receptors may undergo hypermutation, thereby producing high-affinity antibodies. Both the recombination diversity and junctional diversity result in a largely diverse immune repertoire 9. The mechanism for the diversity of the antigen-specific receptors repertoire is shown in the picture description (Figure 1). Traditional methods of analyzing the immune system such as flow cytometry and immunoscope spectratyping are limited and are both labor-intensive and expensive 10, 11.

Before next-generation sequencing (NGS) technologies, namely high-throughput sequencing (HTS), the most common method for profiling immune receptors was the Sanger sequencing 12-14. Nevertheless, there were several drawbacks to using Sanger sequencing: lower data throughput, higher cost, and longer run time 15. Compared with Sanger sequencing, NGS can give a much broader description of the immune repertoire at a significantly lower cost, higher throughput, and a shorter run time. The immune repertoire is affected by the following several problems, such as sample source, errors introduced by PCR amplification and sequencing, and subsequent analysis. In this review, we mainly discussed the methods and challenges of library preparation, sequencing and data analysis. Additionally, we also analyzed the immune repertoire from the single-cell level. Subsequently, we introduced commonly used data analysis tools. Finally, we introduced the application of immune repertoire sequencing in various diseases.

The application of deep sequencing technology enables BCRs and TCRs repertoire to be profiled with higher resolution and larger output. The information from high throughput sequencing of all unique lymphocytes is capable of understanding how several diseases generate, such as cancers, autoimmune diseases and infections. HTS technology was applied to dissecting the immune repertoire variation of patients and healthy individuals 17. Studies have shown that there was poorer diversity of the immune repertoire in rheumatoid arthritis patients than in healthy individuals 18, presumably as a result of the activation of the immune response. There is a disease-specific TCR expressed on the surface of T cells from each patient with lymphoma, which differs from normal T-cells. Targeting the unique CDR3 sequence by chimeric antigen receptor T-cell (CAR-T-cell or CART) therapy was showed to be a viable approach 19. The unique TCR or CDR3 may be used as a biomarker for disease diagnosis, monitoring, and prognosis. There are patient-shared lymphocyte receptor clonotypes, which are closely related to the occurrence of disease 18, 20, 21. These clonotypes may be targets for therapeutic vaccine development or adoptive cell transfer therapy.

## Methodology on adaptive immune repertoire sequencing

### Adaptive immune repertoire sequencing at the population cell-level

**Figure 2:**
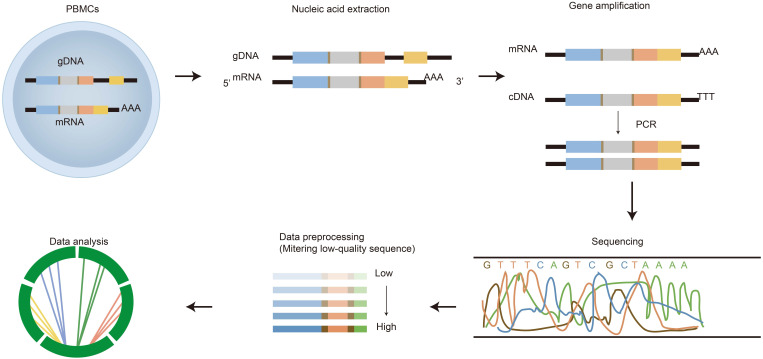
** The process of immune repertoire sequencing and analysis.** The detailed steps of immune repertoire analysis are shown in the figure. The extraction and amplification of samples are the key techniques in library preparation. The starting materials that are either gDNA or mRNA have both advantages and disadvantages as templates. The choice of templates mainly depends on research purposes. gDNA was amplified by multiple pairs of primers, but there were large introns in the DNA, which led to amplification errors. The amplification for mRNA is operated by using 5'RACE which avoids PCR amplification bias, but the operation is more complicated. A large amount of data obtained from high-throughput sequencing of PCR products are used for health assessment, disease diagnosis, monitoring and prognosis.

HTS of the immune repertoire involves several processes shown in Figure 2, including cell handling, nucleic acid extraction, library preparation, sequencing and bioinformatic analysis. The preparation of the library is fundamental for producing a pure library for immune repertoire sequencing. First, raw materials, such as genomic DNA (gDNA) and messenger RNA (mRNA), can be selected as templates for library amplification 22. Choosing which raw materials will depend on downstream goals. gDNA is proportionally associated with the number of cells, thus it is commonly used to calculate the proportion of antigen specificity or target T/B cells; whereas mRNA is closely related to cell function/activation. Both have advantages and disadvantages.

gDNA is easier to obtain and retain because DNA is more stable than RNA 23. Besides, there is no requirement for reverse transcription (RT). Each cell has only one V(D)J gene that has been successfully rearranged, thus gDNA can better reflect the number of cells. However, gDNA requires a higher concentration of starting templates and has a higher affinity for the annealing of primers. Compared with gDNA, mRNA has several advantages. For example, there is a higher number of copies of mRNA in a single cell. Secondly, RNA-seq is capable of producing large information at the gene transcription level. Third, mRNA has no intronless genes, thereby reducing the interference of non-coding signals 24. Finally, the overall length sequence in the CDR region is easily available. Nevertheless, mRNA may introduce errors because of RT 13. In addition, RNA is easily degraded and has relatively high requirements for extraction, transportation, and storage. This greatly increases the difficulty and cost of library preparation, making the cost of RNA-based immune repertoire sequencing higher. In addition, it is difficult to realize the screening and clinical application of large-scale samples. Fortunately, dry blood spots (DBS) were developed to analyze the peripheral blood immune repertoire 25. DBS uses fingertip blood as the sample source, which is simple to operate and low in cost. Studies have confirmed that this method can well avoid RNA degradation and can be transported at room temperature. Most importantly, the diversity of immune repertoire established by this method is similar to that of immune repertoire established by conventional methods, which brings great convenience to library establishment.

It is very important to determine which amplification methods are used for library preparation. Commonly used methods are multiplex PCR (mPCR) and 5'RACE. mPCR uses a mixture of primers to capture multiple regions. In addition, this amplification method can be used for both gDNA and mRNA. The amplification principle is similar to common PCR, except for the increasing number of primers. Unfortunately, there are different efficiency and cross-reactivity among different primers, leading to the introduction of bias in the amplification products. By contrast, 5'RACE is merely applied to capture mRNA rather than DNA. One set of gene-specific primers are able to combine the 3'end gene fragment with its complement. This approach is believed to minimize the bias introduced from the mPCR amplification method 26. However, careful examination of all V gene segments on a locus revealed that some may have short 5'un-transcribed regions (UTR) (as short as 30 bp), and some may have very long 5′UTR (up to 9 kb). Therefore, the libraries made by this method may be biased toward shorter sequences. The set-up and amplification of this approach can be more complex, and its success is highly dependent on the efficiency of reverse transcriptase. In recent years, 5'RACE-PCR and deep sequencing were used to study the immune function of rhesus monkeys for the first time. Several sites containing somatic hypermutation were found in FR3 region 27. In short, the two methods have their own advantages and disadvantages. RNA-seq has also been used to study immune repertoire in recent studies 28. Although it provides more comprehensive gene expression information, this method does not provide the whole transcriptome sequence, which is necessary for identifying low abundance CDR3 sequences in more cells. Therefore, it is not fully sensitive, nor quantitative enough for serious repertoire analysis.

Immune sequence sequencing (IR-seq) is a complex process, which requires well-trained professionals to minimize user errors. Errors can be introduced in many steps, including library preparation and sequencing. Library preparation can introduce errors during nucleic extraction and PCR amplification, whereas sequencing errors stem from different sequencing platforms as well as the concentration and purity loaded to be sequenced. The Roche 454 platform was the first sequencing platform used for IR-seq. Before Roche 454, many other platforms have been introduced into the market, such as Illumina, pacbio, BD and Oxford nanopore. Comparing with Roche 454, Illumina sequencing platforms have many advantages, such as shorter read length, lower cost, and higher throughput. Illumina dominates today's sequencing market 29. Though Illumina instruments have strengths that cannot be ignored compared with the other sequencing instruments, the increased output of shorter read length can produce a higher mismatched sequence 30. Moreover, sequencing by synthesis-based technology introduces substitution errors, while other sequencing platforms, such as Roche 454, Pacific Bioscience, and Ion Torrent, are occupied by insertion/deletions, called indels 29, 31. Substitution errors occasionally occur with base replacements, such as the transversions between nucleotides guanine and thymine (G↔T) or cytidylate and adenine (C↔A) 32. In order to reduce the incorrect base assignment, error correction methods are used, such as clustering algorithms and unique DNA barcoding technology. The purpose of clustering algorithms is to minimize sequencing errors by grouping similar sequences together. The unique DNA barcoding technology is to add unique molecular identifiers (UMIs) which can adjust error or amplification bias in PCR process 33.

**Figure 3:**
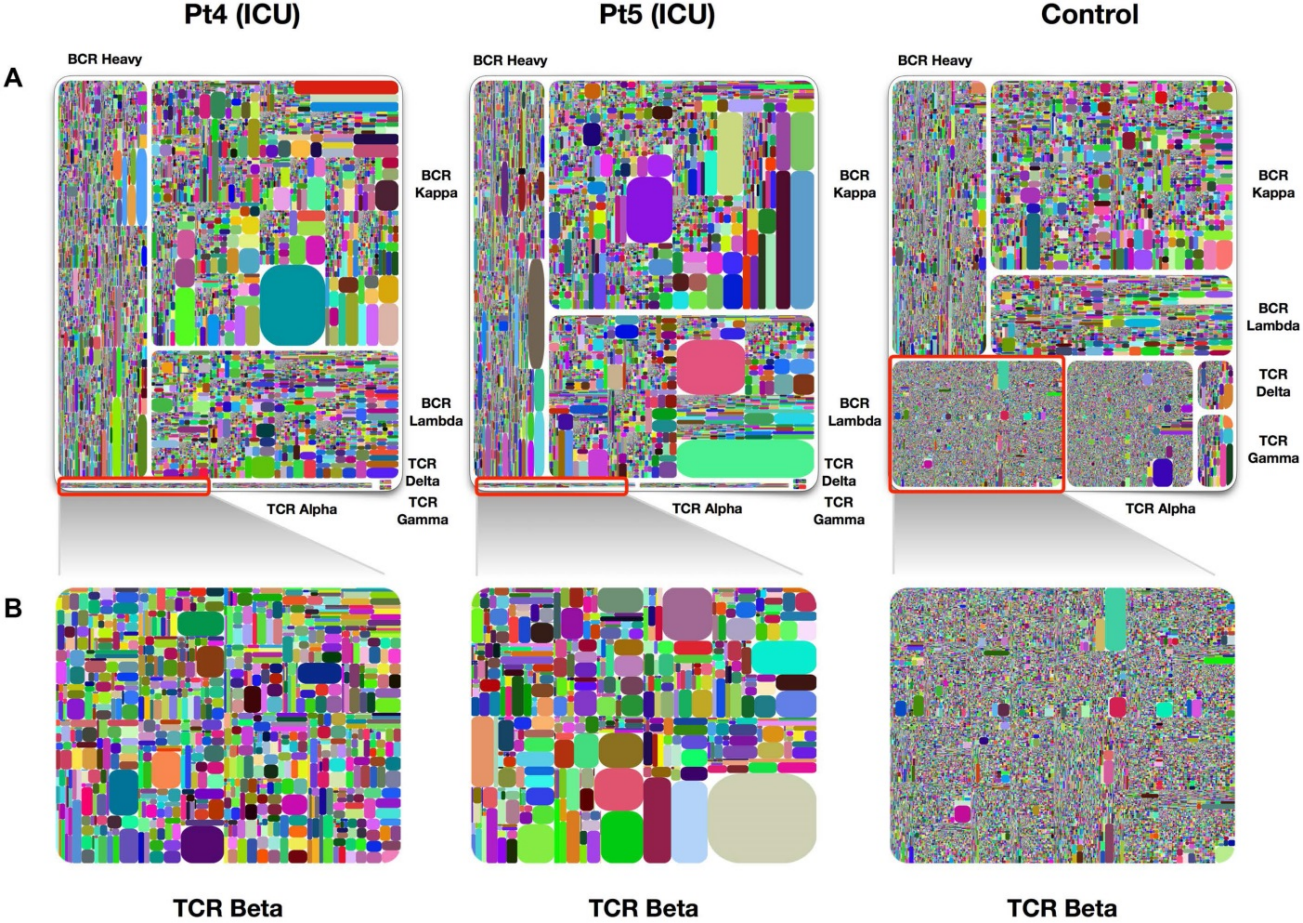
**Treemap of TCR and BCR repertoires in PBMCs of healthy individuals and COVID-19 patients. A.** Treemap of 1 healthy individual and 2 patients, respectively. **B.** Treemap of total TCR Beta chains from patients 4, patient 5, and healthy control. The larger the clones in the picture, the worse the diversity. The diversity of healthy people is better than that of patients. Adapted with permission from Niu, copyright 2020 38. Pt4: patient 4, Pt5: patient 5.

Table 1 listed the current mainstream immune repertoire sequencing products. Adaptive Biotechnologies, iRepertoire, BGI, Illumina, and Thermo scientific are currently several biotechnology companies on the market that focus on adaptive immune system research. They use adaptive immunity to change the diagnosis and treatment of diseases, thus promoting the development of companies in the field of immune driven medicine. Adaptive Biotechnologies has developed a diagnostic product, immunoSEQ which can detect many diseases early through a single blood test. Compared with other platforms, iRepertoire offers both human and mouse immune repertoire research by the addition of UMIs. iRepertoire gives the option to amplify all 7 receptor chains in one reaction. It also has an automated NGS library construction platform. There are many evaluation indicators for the diversity of immune repertoires, such as Shannon, Pielou's evenness, Gini index, and Simpson index. In addition, Diversity 50 (D50) and treemap are also library diversity assessment methods. D50 is a value where the greater the value, the better the diversity. The calculation method is: D50 = (No. of uCDR3 that make up 50% of the total reads × 100)/No. of uCDR3s 25. Treemap is another graph that displays the diversity of the library. It is composed of rectangles with different colors. Different rectangles represent different uCDR3 clones, and the size of the rectangle represents the expression of unique T or B cell clones (see Figure 3).

### Adaptive immune repertoire sequencing at the single-cell level

TCRs and BCRs are composed of multiplex chains (7 chains). Although massively parallel sequencing has become the most common way of sequencing, it can only obtain a lot of information from a large number of cells. It cannot reflect the state and function of a single cell. The reason is that deep sequencing at the population-cell level loses the single-chain pairing information. In order to solve this problem, the endogenous receptor chain pairs were studied by single cell cloning with limited dilution and Sanger sequencing 13. Nonetheless, there are also some disadvantages, such as high cost for sequencing, low throughput, and false pairing of receptors. Therefore, iRepertoire developed iPair single-cell sequencing technology. The iPair single cells sequencing has the ability to describe seven chains from humans or mice at the single-cell level. This technique has a higher throughput, lower cost and faster output. In addition, iPair analyzer is developed by iRepertoire.

Combined with NGS technology, there have been several novel approaches developed to dissect the pairing of B cell heavy: light chains and also T cell alpha: beta or delta: gamma chains. These methods are based on flow cytometry, microfluidic devices, and microwell plates for the isolation of single cells, followed by library preparation through either droplets with unique barcodes or emulsions with magnetic beads 53-57. Mitchell and coworkers identified the public αβ T cell information (i.e. gene usage and paired chains) among sarcoidosis patients by using iRepertoire's PCR, which includes emulsion PCR as well as single-cell PCR, then Illumina sequencing 58. Single-cell separation technology combined with high-throughput sequencing technology helps to achieve high-throughput data and to obtain information on single cells, such as antibody repertoires which is crucial to any disease.

**Figure 4:**
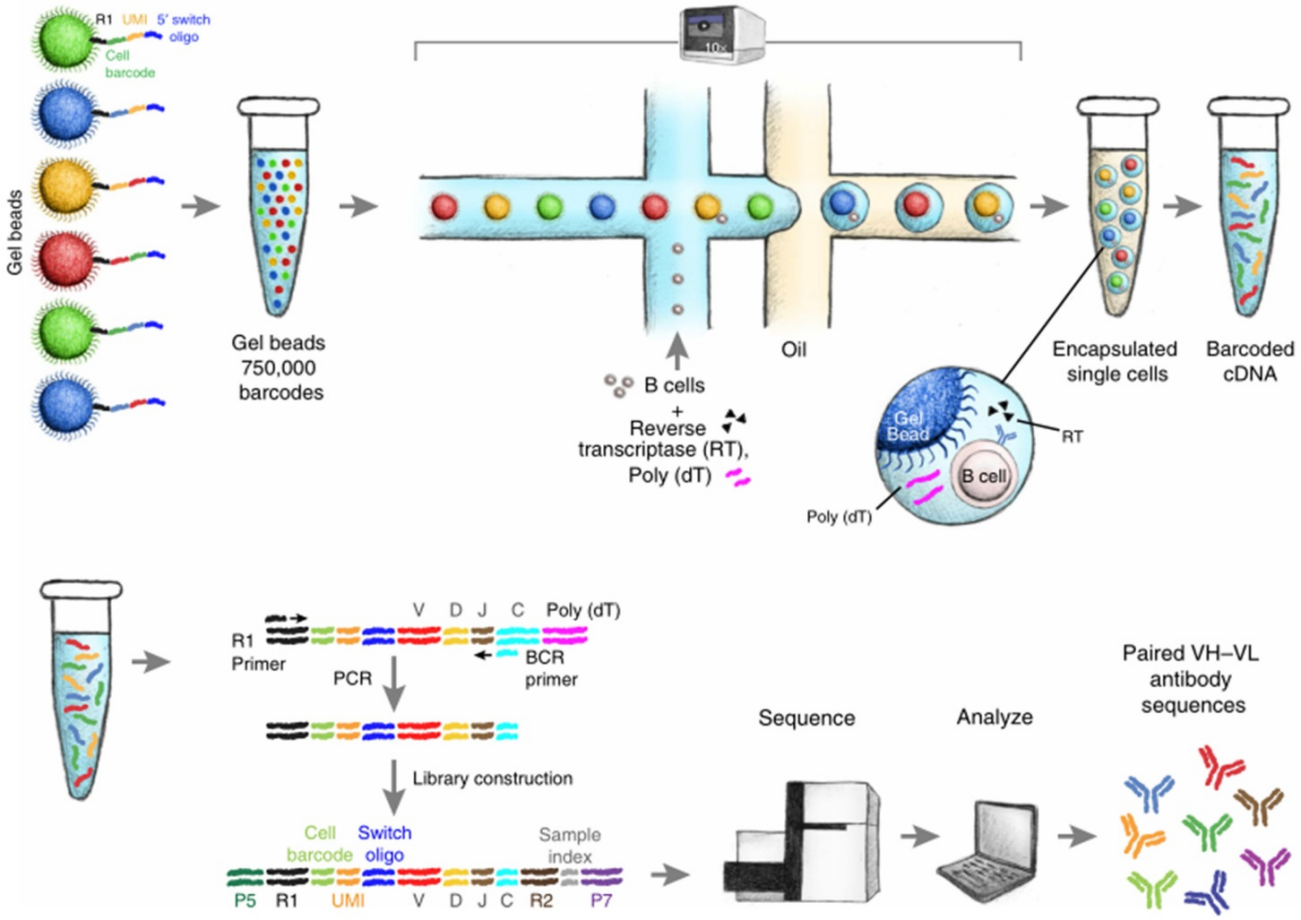
** Schematic diagram of single B cell sequencing.** The magnetic beads with barcodes and UMIs are used to capture individual cells. Magnetic beads and individual cells are coated in oil. Then the cells are lysed and reverse transcription into cDNA. The barcode-encoded cDNA is then reversed transcription into a library and sequenced. The bioinformatic pipeline is used to analyze the heavy and light chain sequences from a single cell. Adapted with permission from Goldstein, copyright 2019 62.

Alternative approaches for analyzing lymphocyte receptors are recently researched to obtain information from the paired chains. For instance, a study from Busse's laboratory combined primer-matrix single-cell PCR (scPCR) and 454 sequencing for antibody repertoire analysis, providing full-length Ig heavy and light chain gene sequence information with UMIs, allowing each sequence to be traced back to the original cell. The tag information requires built-in mechanisms to detect and correct sequencing errors that are prone to traditional NGS methods. In addition, this technique enhanced the output of gene sequences (up to 50,000 single B cells) 59, 60. In 2015, Howie et al*.* reported a novel approach, called pairSEQ, which sorted individual cells into each well in the 96-well plates, followed by reverse transcription into mRNA (specific identifiers are linked to cDNA in this process), amplified library and high throughput sequencing of products 61. Recently, a study by Goldstein et al*.* showed that the establishment of a single-cell receptor sequencing library can quickly discover a variety of antigen-reactive antibodies (see Figure 4). The paired sequences are separated into the same well based on a unique barcode. High-resolution detection of TCR and BCR gene sequences contributes to tumor-infiltrating lymphocytes (TILs), the design of vaccines and the research of autoimmune diseases. If diagnosed at an early stage, most cancers can be relieved and overcome. However, in the early stage, the level of cancer-related biomarkers is low, so it is difficult to detect cancer cells by traditional methods (colonoscopy, X-ray, CT). Therefore, it is necessary to find accurate screening methods to overcome the traditional difficulties of early tumor detection. Strategies using the combination of immune repertoire sequencing technology and bioinformatic analysis were beneficial to identify antigen-specific sequences, thus developing high-quality, patient-specific vaccines.

### Bioinformatic analysis

The meaningful information gained from IR-seq facilitates the understanding of biological meaning. However, the limited information hinders our knowledge of the specificity of BCR/TCR and its clinical application. There is an urgent requirement for bioinformatics methods to profile massive data produced via high-throughput sequencing technology. We have compiled a list of the commonly used open-source software tools for profiling adaptive lymphocyte receptors repertoire sequence data (Table 2). The data processing (see Figure 2) is divided into several steps, including pre-processing analysis, gene assignment, error adjustment, sequence annotation as well as post-processing analysis. There have been tools provided for the first steps consisting of VDJPipe 63 and Presto 64. Heather and colleagues summarized it into two categories: low-level processing and high-level processing 65. Low-level processing includes sequence assembly, VDJ and CDR3 assignment, sequences abundance, and errors correction. High-level processing contains frequency distribution and diversity measurements, VJ usage, sharing and antigen specificity. Others have done similar studies and summarized data analysis tools 66, 67. Although many analysis pipelines are more current for profiling and visualizing immune repertoire sequencing data, several older tools are still common to dates, such as IMGT and IgBLAST. IMGT, the international ImMunoGeneTics database, collects the database of immune globulin, T-cell receptors and major histocompatibility complex (MHC) in all vertebrates. In addition to serving as a data repository like NCBI, IMGT also provides data analysis tools, including IMGT/V-QUEST 68 and IMGT/highV-QUEST 68-70. IMGT/highV-QUEST is more popular due to its higher throughput and ability to replace some common steps of analysis workflow in other data analysis pipelines (i.e. gene identification). IgBLAST was only utilized to dissect immunoglobulin in the early stage, but now it is also available for TCR repertoire analysis through the usage of the IMGT database and the BLAST algorithm 71. Although IMGT and IgBLAST are more commonly used pipelines, more pipelines have been created recently, which are faster, more accurate and more sensitive to the assignment of V(D)J genes and the identification of CDR3. For example, MiXCR/MiTCR is also a commonly used analysis software for profiling IR-seq data 72. It was reported that MiXCR has higher accuracy and faster speed compared to other existing tools because it employs a faster algorithm. Furthermore, this software is easy to install and use, and enables to process PCR errors and indels. Recently, there is increasingly attracting attention for another software tool, namely ImmuneDB 73. ImmuneDB, a novel tool for storing and analyzing data, is recently released. Rosenfeld et al. published an article, which showed ImmuneDB's results were similar to that of MiXCR. Besides ImmuneDB, many tools also support multiple data formats, including AIRR, Change-O 74, VDJtools 75, and Genbank. This analysis tool is not only able to run independently but also combine with other immune repertoire analysis methods. However, there is one limitation: a clone originated from the same individuals or organism requires a uniform V gene sequence, J gene sequence, and CDR3 length. Besides mentioned above, there is a software tool used for the identification of non-regular recombination, such as TRIg 76. Compared with the existing tools, this method is suitable for processing the data generated by RACE amplification, as well as the V (D) J region and the whole gene.

In recent years, new analytical tools (i.e. VDJtools, VDJdb 77, Vidjil 78, and VDJServer 79) have been applied to the analysis of immune repertoire sequencing data. These tools are accessible online, require no installation, and are suitable for researchers and biologists without the knowledge of computers or bioinformatics. Moreover, VDJtools and tcR also offer advanced analysis of TCR repertoire and can process files exported in different formats. VDJdb stores result from T cell assays and couple antigen specificities with TCR sequences. New tools and pipelines are developed every year, further studying each step of the immune repertoire data analysis workflow.

Newly established machine learning (ML) techniques such as DeepTCR, have been made available for parsing and drawing the data generated from IR-seq and contains more precise algorithms. DeepTCR is composed of both supervised and unsupervised learning in T cell repertoires which can predict and describe patterns in data. This method enables the accurate identification of antigen-specific TCRs from a large number of background signals and allows different types of data input 80. immunoML is an open-source ML platform for analysis and classification of adaptive immune receptor repertoire (AIRR), which supports data on B cell and T cell receptors. This software helps researchers select the most appropriate approach to analyze their data by implementing model selection and nested cross-validation 81. Several approaches were used to cluster epitope- or antigen-specific sequences likely to have originated from the expanding sequences of T cell clones. Estimation of the shared antigen-specific TCR sequence can be achieved through multiple algorithms such as TCRdist 82 which permits clustering and visualization, and grouping of lymphocyte interactions by paratope hotspots (GLIPH), which clusters TCRs with high sharing probability 83. The GLIPH algorithm found 141 TCR specificity groups from a dataset of 5711 TCR sequences which were critical for shared-antigen recognition. Another ML framework, DeepRC, was founded off modern Hopfield networks, and was found to outperform all other test methods in terms of predictive performance and could extract sequence motifs related to a given disease category 84. Compared with the Shannon entropy, where sequence relatedness was masked by diversity, ImmunoMap analyzed both frequency and relatedness of TCR sequences and was able to fully recognize the homologous responses of both self-antigens and foreign antigens. ImmunoMap was found to be more effective in finding clinically relevant and predictive TCR labels in tumor treatment than the Shannon entropy 85. A recent study shows a new ML approach that combined standard ML to mine full repertoire data. This approach allowed researchers to represent k-mers as biochemical features and predicted a label for the whole repertoire, thus presenting statistical classifiers of immune repertoire data 86.

## Advances of IR-seq in clinical application

The progress made in high throughput sequencing technology has enabled novel methods for studying the adaptive immune system. IR-seq refers to the use of 5' RACE or mPCR to amplify CDR region of TCRs or BCRs, followed by high-throughput sequencing. Immunogenomic sequencing is commonly used to study the diversity of the adaptive immune system, as well as the relationship between immune cell receptors and diseases, which are crucial for the discovery of biomarkers and drug discovery.

### Minimal residual diseases

The research and characterization of lymphatic hematological tumors have become an increasingly important field in recent years. This disease mainly stems from T or B cell lymphoma and leukemia. Although the treatment for this disease shows good remission, the disease is prone to relapse due to the existence of a small number of cancer cells that cannot be detected using conventional methods. The quantification of minimal residual disease (MRD) plays a key role in prognosis and has become a major identifier. Previous studies showed that MRD information was gained through profiling a large number of the adaptive immune receptor repertoire from healthy individuals and patients 53, 88. Combined with molecular recognition technology, we can accurately evaluate the number of leukemia cells, judge whether diseases will relapse or not, and guide drug use. Nowadays, the number of MRD deciding whether one is likely to relapse has been known (i.e. 10^-2^ or more, 10^-3^, and 10^-4^ or less). Once the amount of MRD is more than the threshold value (10^-4^) following stem cell transplantation, then the relapse of acute lymphocytic leukemia (ALL) can be predicted 89. Therefore, monitoring of MRD using these described methods can provide an early treatment intervention, and conduct the development of novel therapeutic drugs 90-92.

Several commonly used measurements have been developed to determine MRD, such as allele-specific amplification combined with real-time quantitative PCR (ASO-qPCR), flow cytometry, and high-throughput sequencing technology 93. ASO-qPCR and flow cytometry have a high sensitivity ranging from 1×10^-4^ to 1×10^-5^(one leukemia cell in every 100000 leukocytes) 54. However, the ASO-qPCR for profiling immunoglobulin and T-cell receptor genes has several limitations, e.g., high cost, time-consuming and lacking a unified model. These disadvantages impose restrictions on ASO-qPCR. In addition, though flow cytometry is available, the lower sensitivity (which does not reach 10^-4^) is not suitable for probing MRD during and after treatment. Therefore, the assessment of MRD remains difficult in highly heterogeneous malignant lymphoma or lymphocytic leukemia. The advancement of HTS creates many insights into the detection and prognosis of diseases. Therefore, novel higher throughput approaches, such as HTS and next-generation (multidimensional) flow cytometry, are developed to address these problems 94. Compared with the first two strategies mentioned above, HTS and next-generation flow cytometry for MRD quantification have higher sensitivity ranging from 0.001% to 0.0001% and lower false-negative rate 16, 95-97. Subsequently, multiparameter flow cytometry and HTS are proved to be complementary roles.

HTS of BCR/TCR repertoire can analyze the gene rearrangements, thus discovering clones in hematological malignancies in B and T cells. Prior works demonstrated the clonal rearrangement of IgH and TRB in B and T malignant cells, which derived from either bone marrow or peripheral blood samples. Due to the acquisition of bone marrow is more traumatic and invasive, a question was put forward whether peripheral blood (PB) sample can replace bone marrow (BW) sample. Only in T cell acute lymphoblastic leukemia (T-ALL), BM sample might be similar to PB sample 98. Because the distribution of T cells in BM was comparable to that in PM. However, the results stemmed from bone marrow are more preferential than that of the peripheral blood sample in B cell acute lymphoblastic leukemia (B-ALL). Because bone marrow is the source of B cells. The evidence showed that the clonal rearrangement was observed by massively parallel high-throughput sequencing IGH, and confirmed that leukemic gene clones could be identified by using peripheral blood samples 99. Therefore, with the progress of science and technology, MRD detection will be more efficient, convenient, and sensitive.

Overall, IR-seq combined with UMI and bioinformatic tools provides novel insights for checking MRD and drives the rapid advent of prognostic factors 100. The bioinformatic analysis and visualization software were used to process several data generated from IR-seq 101. Compared with other published software, iRepertoire iRweb analyzer provides a comprehensive analysis for 7 chains covering BCR-heavy, - kappa, and -lambda chains as well as TCR- alpha, -beta, -gamma, and - delta chains, from human and mice. With the advancement of bioinformatics pipelines, future studies regarding MRD in lymphoma or multiple myeloma will be further probed with high-resolution methods. The combination of these tools mentioned above contributes to the understanding and management of lymphatic hematological tumors and promotes the development of new and improved treatments. These measures reveal the relationship between MRD assessment, clinical outcome, and the assessment of treatment response.

### Homologous stem cell transplantation

The approach of IR-seq in transplantation mainly includes hematopoietic stem cell transplantation (HSCT) and organ grafting. The former is an effective method for relapsed or refractory diseases. The latter is commonly used for assessing rejection risk after organ transplantation. Monitoring the diversity of adaptive immune repertoire pre-and post-transplant can avoid infections and relapse. Therefore, it is noted that the focus needs to be on the diversity of TCR and BCR repertoires following HSCT or chemotherapy. Clinical trials have demonstrated that hematopoietic stem cell transplant recipients promoted cancer remissions and the recovery of immune repertoire diversity 102, 103. Researchers performed an assessment of patients with severe multiple sclerosis (MS) after HSCT. The results showed that the patients who received treatment had renewed the immune repertoire to make it similar to the situation before treatment 35, 104.

Delayed immune reconstruction and infection after hematopoietic stem cell transplantation are the main causes of its morbidity and mortality. Therefore, monitoring immunological reconstitution or the diversity of the lymphocyte receptors repertoire is a major strategy for disease prevention. However, the lymphocyte cell repertoire is so complex that it is difficult to analyze the overall repertoire. Hence, the evaluation of immune repertoire with high-resolution approaches is of utmost importance for patients after hematopoietic stem cell transplantation. Over the last two decades, there have been several methods developed to explore immune receptors repertoire. One strategy is flow cytometry, used to determine the existence of diverse immune receptors families. PCR is another strategy that aims to profile the usage of variable region genes in TCRs and BCRs. The CDR3 size spectratyping is regarded as the third strategy, measuring the changes of length in the CDR3 region. However, these methods are unable to detect the frequency of individual receptors and can only evaluate the complexity of the immune repertoire.

In recent years, deep sequencing of the T and B cell receptors has been possible. HTS has been used to analyze immune reconstruction and exhibits unprecedented advantages 105, 106. Compared with conventional ways, HTS improves the precision of TCR and BCR frequency. For the past few years, literature particularly focused on the quantitative characterization of immune cell repertoire in HSCT recipients 107, 108. Utilizing 5'RACE and deep sequencing technology amplified TCRs from peripheral blood and cord blood-graft recipients showed that the diversity of TCRs repertoire conclusively recovered and the TCR diversity of recipients from cord blood-graft was similar to that of healthy individuals. However, cord blood transplantation has attracted more and more attention due to the low risk of acute chronic graft-versus-host disease (GVHD) 109.

Even though HSCT is a key method to treat leucocythemia and immunodeficiency disease 110, GVHD is a serious problem after HSCT, which determines graft success or failure. A study early reported that regulatory T cells had the ability to prevent GVHD by banning the expansion and function of common T cells. Recently, selective T cells -depleted hematopoietic stem cells show an effective approach to decrease graft-versus-host disease 111, 112. Therefore, selective T cells depleted following HSCT could be widely used for clinical evaluation in the future.

### Autoimmune diseases

Immune cells not only attack foreign pathogens but also autologous antigens, leading to autoimmune diseases. It consists of multiple diseases, which cause the body's immune response to self-antigens (organs and cells), thus resulting in damage to self-tissues and immunodeficiency. Autoimmune diseases have multiple organ involvement, often leaving the pathogenesis unclear 113, 114. Deep sequencing of the immune repertoire of patients with autoimmune diseases can provide important mechanism analysis, even potentially contributing to treatment. In 2010, the TCRs and BCRs repertoire in synovial tissue and peripheral blood from patients were profiled for the first time utilizing deep sequencing 21. Data showed the distinct difference in immune repertoire diversity between synovial tissue and peripheral blood. Subsequently, studies demonstrated that actionable biomarkers, which were used for diagnosis, prognosis, and selection of therapies in clinical practice, were detected by HTS of BCRs and TCRs 115-117. For instance, by analyzing TCR repertoire variations in systemic lupus erythematosus (SLE) patients 118, RA patients 119, 120, and healthy controls, Liu et al. identified TCR clones which related to autoimmune diseases 39. However, these clones were decreased after therapy. That said, these clones can serve as markers for tracking the immune response associated with autoimmunity 44.

Recent researches also reveal that the V gene repertoire decreases within diseases. Studies have shown that the HLA-DR15 haplotype was related to increased self-reactivity in multiple sclerosis (MS), and memory B cells mediated their proliferation in an HLA-DR-dependent manner. Anti-CD20 targeting B cells could effectively deplete B cells and reduced the proliferation of T cells 49, 121. The researchers used an unbiased epitope discovery method to identify RASGRP2 as a target autoantigen expressed in the brain and B cells 49. RASGRPs are involved in the reduction of apoptosis and tumorigenesis. These findings will help solve important problems related to the interaction of pathogenic B-T cells in a variety of diseases, and may also develop new therapies. Additionally, the public clones are presented among different patients, and the sharing sequences have shorter gene fragments 36, 50. It is worth noting that regulatory T (Treg) cells play an important role in controlling autoimmune diseases, such as type 1 diabetes. A study by Spence et al 122 showed Treg cells clonotype expansion in islets from non-obese mice with diabetes, which was driven by specific antigens (i.e. insulin B:9-23 and proinsulin). In recent years, studies have confirmed that CD5^+^ B cell expansion exists in many autoimmune diseases, mainly including SLE, RA, and type 1 diabetes 123. Moreover, CD5^+^ B cells are able to produce natural antibodies. These B cells may contribute to autoimmune clues which can provide new information about autoimmune disease.

Progress has been made in treating autoimmune diseases in recent years. However, novel effective treatments remain to be developed. Therefore, we raise these questions: 1) whether another pathway exists or not and is likely to generate autoantibodies; 2) whether the expanded clones will continue after stopping therapy. Conclusively, the acquisition of etiology and pathogenesis is further learned by IR-seq.

### Infectious diseases

High throughput sequencing technology is a powerful tool for uncovering changes in immune system composition caused by infectious diseases. IR-seq is capable of providing in-depth insights into antibody response to vaccination or pathogen stimulation, which affect naive immune receptors repertoire. Therefore, numerous studies have attempted to dissect adaptive immune receptor repertoire following antigen challenge. The pre-existing immunity related to antigens exposure history has an important impact on antibody response to vaccines. For instance, George et al. have attempted to unlock molecular mechanisms of antibody response to vaccines through profiling of serum antibody repertoire following vaccination 124, 125. This approach indicated that memory B cells were activated or recalled. In addition, antigen exposure leads to a decrease in B cell repertoire due to the deletion of lymphocytes. Cheng et al. characterized the profile of γδ T cells repertoire in pulmonary tuberculosis patients at sequence resolution for the first time using NGS technology 126. Studies of BCR and TCR repertoires have spurred the recent interest in designing universal vaccines. Interestingly, the immune repertoire of patients who were infected by Coronavirus Disease 2019 (COVID-19) was reported in 2020 38. The results showed that the TCR repertoire decreased in the early stage of onset, while the BCR and TCR repertoire increased during the recovery period. Therefore, the TCR repertoire can be used as a predictive marker of disease. In this study, the changes of the seven chains repertoire including α-β TCR, γ-δ TCR and BCR-IgH, -IgK, -IgL in the early stage of COVID-19 infection were reported for the first time. Adaptive Technologies recently partnered with Microsoft to create a platform called ImmuneRACE 127. This platform, combined with the ImmuneCODE database, was used to study people who were infected with the SARS-CoV-2 virus, those who have recovered from the infection, or close contacted, to identify disease-specific immune signatures, and further deepen the understanding of the adaptive immune response to the SARS-CoV-2 virus. The potential of the immune repertoire in predicting disease infection is confirmed.

A vast array of information from sequencing hypervariable regions can be used for diagnostic biomarkers in infectious diseases. Even though evidence has accumulated that the adaptive lymphocyte repertoire is complicated and diverse, there is a commonality between different individuals. Notably, the public antibody clones have been confirmed to exist on multiple HIV-infected individuals by analysis of the antibody repertoire 128. It has been reported that the shared clones or overlapped sequences derived from diverse patients can be served as biomarkers for diagnosis 129, 130. The detailed characterization of antibody repertoire may be beneficial to vaccine evaluation. Considering the rapidly evolving Ebola virus, researchers found that a small number of monoclonal antibodies contained all neutralizing monoclonal antibodies 131. IR-seq of V genes or CDR3 from antibodies helps to uncover antigen lineages.

Utilizing IR-seq can help researchers answer why the elderly are vulnerable to infection and have difficulties responding to vaccinations. Immune repertoire sequencing has become an effective method for the diagnosis, prognosis and vaccine design of infectious diseases. The immune diversity is age-dependent, indicating that immunologic function declines with increasing age 45, 132, 133. However, it is surprising that murine cytomegalovirus (MCMV) has been proposed to contribute to immune defense in older mice 51. Moreover, infants are most vulnerable to infection as their immune systems are not fully developed 52. However, current studies on the diversity of immune repertoire of young children infected with malaria have found that their immune repertoire is diverse, although they do not accumulate as many mutations as adults 134. The changes of the immune system of the elderly and the antibodies in the sera of infected patients were studied in detail, which contributed to the development of vaccines 135-137.

Antigen-specific monoclonal antibodies can be identified by single-cell sequencing. Monoclonal antibodies can be used as a diagnostic and therapeutic approach. Coelho reported the antibodies produced during malaria infection or vaccination, which provided information for vaccine development 138. They tested nine pairs of heavy and light monoclonal antibodies, one of which was identified as a neutralizing, transmission-blocking antibody. With the development of single-cell pairing technology, more and more therapeutic antibodies are used to treat infectious diseases.

### Cancer immunotherapy

Immunotherapy is divided into 5 different classes: cell-based immunotherapies, immunomodulators (checkpoint inhibitors), vaccines, antibody-based targeted therapies and oncolytic viruses. Traditional treatment methods (radiotherapy and chemotherapy) have a poor curative effect due to tumor resistance to treatment 139. The role of immunotherapy is to enhance or inhibit the immune system to fight against diseases, and kill cancer cells and tumor tissues relying on the immune function. Recently, scientists found that targeting macrophages may be a potential treatment and summarized the role of macrophages in tumors 140. However, tumor heterogeneity creates a major challenge with immunotherapy, including low response rate and drug resistance limitations. The emergence of IR-seq may help to understand the underlying molecular mechanism, and to develop personalized medicine.

Antigen-specific T cell monitoring is of the utmost importance for immunotherapeutic research. Klinger et al developed a new assay, MIRA (Multiplexed Identification of T cell Receptor Antigen Specificity), combining standard immune assays with IR-seq to identify antigen-specific T cells 141. Danilova's study also presents a new assay to identify antigen-specific cells, namely FEST (Functional Expansion of Specific T cells) 142. FEST overcomes the disadvantages of traditional antigen dection assays by using TCRseq to analyze antigen-specific clonal expansion in combination with a bioinformatics platform to detect antigen-specific T cells with higher sensitivity, specificity and throughput.

**Figure 5:**
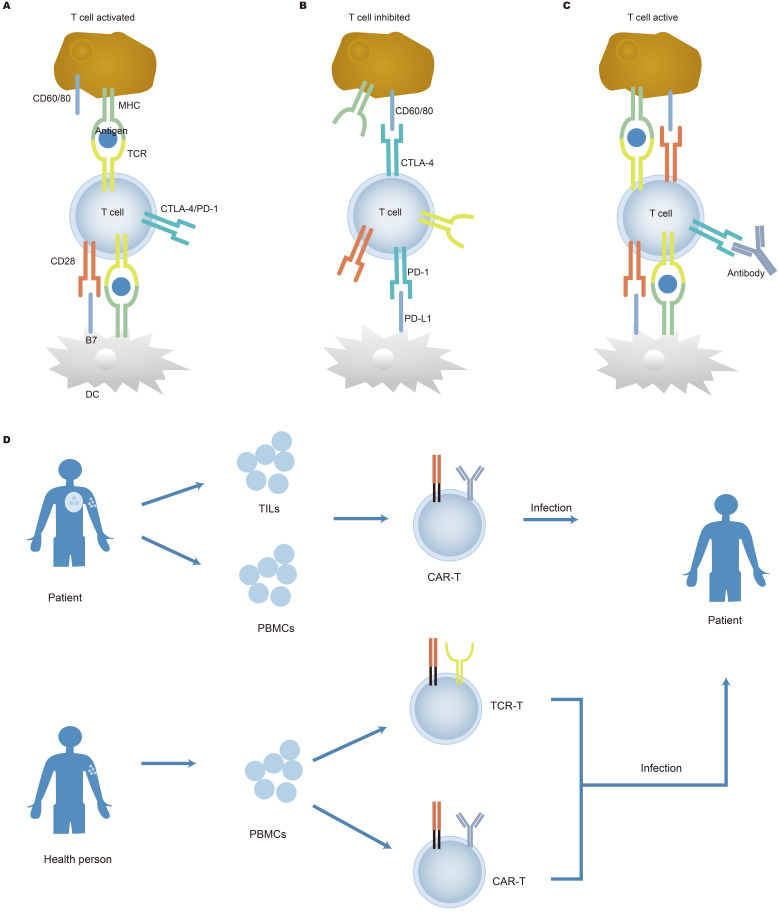
** Cancer immunotherapy**. The function of immune checkpoint and treatment are shown in figures **A, B, and C.** Co-stimulatory and co-suppressive receptors which include CD28, PD-1, and CTLA-4 are expressed on the surface of T cells. T cells activate and initiate an immune response when it accepts a stimulus signal; otherwise, T cells inactivate when the suppression signal is received, the T cell immune response is suppressed. Therefore, the target of the inhibitor is to block a combination of the ligands on tumor cells and its receptors, thereby activating T cells response to cancer cells. Nevertheless, the therapeutic effect of checkpoint inhibitors varies from person to person. Adoptive cell therapy is demonstrated in Figure 5D. The figure shows the cell source, isolation, optimization, and reinfusion process of adoptive cell therapy. TILs from patients, T cells in peripheral blood of patients, and peripheral T cells of healthy people are cultured *in vitro*, optimized, expanded, and then reinfused into the human body to increase accurate antigen recognition and enhance the lethality, making it possible to improve the treatment of tumors. APC: antigen presenting cell; DC: dendritic cell.

IR-seq has been used to check the dynamic changes of the immune repertoire before and after treatment. Immune checkpoint inhibitors work by preventing ligands bind to receptors on the surface of immune cells, thus allowing T cells to kill cancer cells. Major immune checkpoint are CTLA-4, PD-1, and CD28 46, 47, 143, illustrated in Figure 5A, 5B, and 5C. The mechanisms of the immune checkpoint inhibitors in cancer immunotherapy have been further elaborated in recent studies 144. In one study, patients were treated with surgery and PD-1 inhibitor nivolumab. After 2-4 weeks of treatment, an increase in antigen-specific T cell clones was observed, and these clones were not detected without nivolumab treatment, further proving that PD-1 blockade promoted intratumoral T cell killing and enhanced tumor antigen-driven priming of T cells 145. The immune repertoire is a potential biomarker that can reflect the immune response caused by immunotherapy. It was reported that after the immune checkpoint was blocked, new cells were observed in patients, while no significant changes were observed in healthy people 37, 146. This increase indicated that the immune system was activated after treatment. Generally, patients with high PD-L1 expression, high tumor mutations, and mismatch repair defects can predict immunotherapy responses that are blocked by immune checkpoints 147. However, Smith observed the lasting clinical benefit in two patients lacking the above biomarkers and detected specific T cells in the peripheral blood of the patients after treatment 147. On the other hand, TCR sequencing showed that in patients with advanced melanoma treated with ipilimumab, the expansion of T cell clones was observed before and after nivolumab treatment 148. Although checkpoint blockade alone can play a major therapeutic effect, combined with target therapy may enhance the therapeutic effect 149, 150. Another study related to immunotherapy showed that patients with increased T cell proportions after treatment had an increased likelihood of survival 151. Poran et al. showed that the use of immune checkpoints combined with vaccines to treat patients with melanoma 152. The results revealed that TCR repertoire diversity and stability increased in patients with progression-free survival. There is a positive correlation between clonality and stability, so immune repertoire sequencing can accurately assess immunotherapy response. Other studies have also shown that the immunological library was highly predictive of checkpoint blocking therapy, indicating the reliability of the immune repertoire as a biomarker 50, 153. A combination of checkpoints with other treatments had been proved a great clinical outcome in early-stage breast cancer 154-157. Radiation therapy can increase the ratio of CD8/CD4 when used alone. When radiotherapy combined with anti-checkpoint blockade, T cells showed clonal proliferation. However, only a subset of patients who received treatment showed tumor remission. It was speculated that the benefited patients may have an inert anti-tumor response.

Adoptive T cell therapy (Figure 5D) has made significant progress in curing hematological malignancies. It has been reported that tumor-infiltrating lymphocytes (TILs) proliferated *in vitro* stimulated by cytokines (such as IL-2) and were refused into the body of patients, which contributed to the remission of cancer. TILs isolated from tumor patients can recognize tumor mutations which result in the generation of heterogeneous tumors 158, 159. This breakthrough clinical research achievement has also brought new inspiration to chimeric antigen receptor T cell (CAR-T) therapy for solid tumors. Deep sequencing of TILs reveals information on intra-tumor heterogeneity and clonal distribution. Simultaneously, a study by Yan et al. showed low TCRs sequence reads within tumors, indicating that T cell response to tumors in retroperitoneal liposarcoma was low 160. Increasing evidence has shown that the diversity of TCR repertoire in TILs differs from that of TCR repertoire from both adjacent healthy mucosal tissue and peripheral blood 161, 162. Therefore, the detection of TCRs expressed on the surface of TILs has the potential to be used as a biomarker and may offer therapeutic targets. Engineered cells play an important role in cancer treatment, specifically in CAR-T cells. The application of CAR-T cells has displayed remarkable clinical efficacy in hematological malignancies 163, 164. After the infusion of anti-CD19 CAR-T cells, clonal expansion of T cells was observed in patients with chemotherapy-refractory and CD19^+^ diffuse large B-cell lymphoma (DLBCL) 165, 166. After the engineered cell therapy, the T lymphocytes repertoire was restored, indicating that changes in the T lymphocytes repertoire can predict disease progression. In addition, there are new targets for targeting CD22 and CD30 CAR-T for the treatment of tumors. Dual-target CAR-T infusion therapy for patients with multiple myeloma has a total response rate of 95%, of which 85% of patients did not observe recurrence during treatment, indicating that dual CAR-T therapy may become a prominent method of treating cancer 167. Although adoptive T cell therapy benefits patients, side effects are also a problem that cannot be ignored. Because cancer cells are similar to normal cells, normal cells can also be attacked by the therapy.

Vaccine therapy activates immune cells to attack tumor cells. A study showed that the six patients were vaccinated with neoantigens, the cancer recurrence was not observed among four individuals 25 months after vaccination; the remaining 2 patients showed complete tumor regression after anti-PD-1 treatment, indicating that novel T cells specific for tumors were expanded 168. After cancer patients received immunotherapy, results showed that the survival of patients with effective response was significantly prolonged, and immune diversity was increased 169. Dendritic cell immunotherapy has shown exciting results in the treatment of gliomas. There was a significant correlation between the increase of TILs levels and the increase of overall survival rate after treatment, and the degree of TCR sequence sharing was related to the improvement of clinical outcomes 170. This result implied that the immune repertoire could predict the effect of cancer vaccine treatment. Since tumor antigens are very similar to normal cell antigens, vaccine therapy may also cause adverse side effects. Cancer heterogeneity which is caused by an accumulation of genetic alterations leads to the requirement to develop personalized cancer vaccines. This has been made possible with the development of tumor gene sequencing techniques. Subsequently, the successful application of individualized tumor vaccine in clinical trials proved that it is feasible and safe, and has immunotherapy targeting for individual tumor mutation characteristics 171-175. IR-seq is used to assess the effectiveness of vaccines, select therapeutic targets, and cancer prognosis.

## Conclusions

IR-seq has changed our knowledge of host immune response to a diverse range of internal and external pathogens, especially revealing a myriad of specific receptors repertoire that can target antigens epitope by paratope. This technology has revealed good prospects in various disease research, such as autoimmune diseases, infectious diseases, and tumors. Diagnosis, monitoring, and treatment of tumors have always been the focus of scientific research. Since the success of checkpoints in melanoma, immunotherapy for cancer has rapidly advanced. In this review, we summarized the adaptive immune receptor repertoire sequencing methods and the progress that has been made. With the advancement of high-throughput sequencing technology, the study of gene sequences overcomes the previous shortcomings that only dozens of sequences could be studied. Even though high-throughput sequencing can analyze large sample sizes at one time, only partial information can be obtained. However, single-cell technology can accurately study the nuances of individual cells. The single-cell pairing has allowed researchers to discover more therapeutic antibodies, which can be used in the treatment of cancer, autoimmune and infectious diseases. The combination of high-throughput technology and single-cell technology not only reduces costs but also increases throughput, which will provide new insights into the development of tumor vaccines. Moreover, high-throughput sequencing technology turns the problem from data acquisition to data management and analysis. If we want to get a lot of information associated with immune response, then a detailed experimental design and large-capacity data processing tools are required. In addition, a diverse range of data requires a universally standardized algorithm to be processed.

There are many studies on immune receptor repertoires 48, 176, 177. However, there are still some limitations that are worth studying, such as the starting materials. Although lymphocyte cells from peripheral blood make up only 2% of the total lymphocyte cells 13, peripheral blood is attained to study lymphocyte receptor libraries due to the ease of collection. Other sources (i.e. lymph nodes, spleen, mucosal surfaces, red bone marrow) are traumatic and invasive, which make collection difficult. It is worth noting that the quality of the primers determines whether the library can reflect the true state of the organism. Over the years, studies on previously, IR-seq was mainly focused on BCR heavy and light chains, TCR alpha and beta chains, and a few TCR gamma and delta chains. In recent years, more and more attention has been paid to the diversity of CDR3 regions of gamma and delta T cells, and the impact of these cells on intestinal flora 178, 179. With the improvement of IR-seq technologies, researchers may greatly expand the application of immunological library sequencing in other diseases allowing for more advanced therapies and cures.

## Figures and Tables

**Table 1:** Mainstream immune repertoire sequencing productions

	Adaptive Biotechnologies	iRepertoire	BGI	Illumina	Thermo scientific
Production	ImmunoSEQ	iR-Harmony	BGISEQ-500	Illumina HiSeq/MiSeq	Ion AmpliSeq Immune Repertoire
TCR/BCR	TCR	TCR/BCR	TCR/BCR	TCR/BCR	TCR
Receptor type	β	All (7 chains)	α/β	α/β	β
DNA/RNA	DNA/cDNA	DNA/RNA	DNA/RNA	DNA/RNA	RNA
Human/Mouse	Human, Mouse	Human, Mouse	Human	Human	Human
Amplification method	Multiplex PCR	Multiplex DAM- PCR	Multiplex PCR	Bridge PCR	Multiplex PCR
UMIs	No	Yes	No	Yes	No
Ref.	34-37	25, 38-40	21, 41-44	14, 17, 45-48	49- 52

**Table 2:** Common information analysis tools

Tools	File format	TCR/BCR	Run	Programming languages	Maximum input value	Sequence quality	UMIs	Clustering	Ref.
IMGT/HighV-QUEST	FASTA	TCR/BCR	Online	-	150000	NO	NO	Yes	68-70
IgBLAST	FASTA	TCR/BCR	Online/stand-alone	C++	<1000	NO	NO	NO	71
VDJPipe	FASTA	TCR/BCR	Online/stand-alone	C++	None	Yes	Yes	NO	63
MiXCR	FASTA/FASTQ	TCR/BCR	stand-alone	Java	None	Yes	NO	Yes	72
ImmuneDB	FASTA/FASTQ	TCR/BCR	stand-alone	Python	None	Yes	Yes	NO	73
VDJtools	FASTA/FASTQ	TCR	stand-alone	Java/Groovy	None	NO	Yes	Yes	75
VDJServer	FASTA/FASTQ	TCR/BCR	Online	-	None	Yes	Yes	Yes	79
VDJdb	FASTA/FASTQ	TCR	stand-alone	Java	None	Yes	NO	NO	77
TRIg	FASTA	TCR/BCR	stand-alone	Perl	None	NO	NO	NO	87
Vidjil	FASTA/FASTQ	TCR/BCR	stand-alone	C++/Java	None	NO	NO	Yes	78

## Abbreviations

CDR: complementarity determining regions
TCR: T-cell receptor
BCR: B-cell receptor
NGS: Next-generation sequencing
HTS: high-throughput sequencing
gDNA: genomic DNA
mRNA: messenger RNA
RT: reverse transcription
DBS: dry blood spots
mPCR: multiplex PCR
RACE: rapid amplification of cDNA ends
UTR: un-transcribed regions
IR-seq: immune repertoire sequencing
UMIs: unique molecular identifiers
D50: diversity 50
scPCR: single-cell PCR
TILs: tumor-infiltrating lymphocytes
MRD: minimal residual disease
PB: peripheral blood
BW: bone marrow
ALL: acute lymphocytic leukemia
T-ALL: T cell acute lymphoblastic leukemia
B-ALL: T cell acute lymphoblastic leukemia
HSCT: hematopoietic stem cell transplantation
MS: multiple sclerosis
GVHD: graft-versus-host disease
RA: rheumatoid arthritis
SLE: systemic lupus erythematosus
PD-1: programmed cell death protein 1
CTLA-4: cytotoxic T lymphocyte-associated antigen-4
CAR-T: chimeric antigen receptor T cell
DLBCL: diffuse large B-cell lymphoma
